# Chilblains

**Published:** 1860-04

**Authors:** 


CHILBLAINS — MR. DUCHESNE-DUPARC'S FORMULA. — Mr. Duchesne-Duparc has kindly forwarded to us his receipt for chilblains, with some remarks on the treatment of this distressing affection:

℞ Liq. calcis, 4 oz.; liquoris ammoniæ, 1 ʒ; spirit. minth. 4 ʒ; tinct. saponis, ʒ 1. M. For embrocation night and morning, on the parts affected.—*Champonniere's Journal.*

